# Osteoporosis in Frail Patients: A Consensus Paper of the Belgian Bone Club

**DOI:** 10.1007/s00223-017-0266-3

**Published:** 2017-03-21

**Authors:** E. Gielen, P. Bergmann, O. Bruyère, E. Cavalier, P. Delanaye, S. Goemaere, J.-M. Kaufman, M. Locquet, J.-Y. Reginster, S. Rozenberg, A.-M. Vandenbroucke, J.-J. Body

**Affiliations:** 1 Gerontology and Geriatrics, Department of Clinical and Experimental Medicine, KU Leuven & Center for Metabolic Bone Diseases, UZ Leuven, Herestraat 49, 3000 Leuven, Belgium; 2 Department of Radioisotopes, CHU Brugmann, Université Libre de Bruxelles, Bruxelles, Belgium; 3 Department of Public Health, Epidemiology and Health Economics, University of Liège, CHU de Liège, Liège, Belgium; 4 Department of Clinical Chemistry, UnilabLg, CIRM, University of Liège, CHU de Liège, Liège, Belgium; 5 Department of Nephrology, Dialysis, Transplantation, University of Liège, CHU de Liège, Liège, Belgium; 6 Unit for Osteoporosis and Metabolic Bone Diseases, Ghent University Hospital, Ghent, Belgium; 7 Department of Endocrinology, Ghent University Hospital, Ghent, Belgium; 8 Department of Gynaecology-Obstetrics, Université Libre de Bruxelles, Bruxelles, Belgium; 9 Clinical Department of Internal Medicine, UZ Leuven, Leuven, Belgium; 10 Department of Medicine, CHU Brugmann, Université Libre de Bruxelles, Bruxelles, Belgium

**Keywords:** Osteoporosis, Frailty, Cancer, Dialysis, Elderly, Sarcopenia, Anorexia nervosa

## Abstract

In this consensus paper, the Belgian Bone Club aims to provide a state of the art on the epidemiology, diagnosis, and management of osteoporosis in frail individuals, including patients with anorexia nervosa, patients on dialysis, cancer patients, persons with sarcopenia, and the oldest old. All these conditions may indeed induce bone loss that is superimposed on physiological bone loss and often remains under-recognized and under-treated. This is of particular concern because of the major burden of osteoporotic fractures in terms of morbidity, mortality, and economic cost. Therefore, there is an urgent need to appreciate bone loss associated with these conditions, as this may improve diagnosis and management of bone loss and fracture risk in clinical practice.

## Introduction

Life expectancy has increased substantially over the past centuries. Along with the aging of the population, the incidence of age-associated conditions increases and more attention and resources will be needed to manage these conditions. Osteoporosis, characterized by low bone mineral density (BMD) and microarchitectural deterioration of bone tissue associated with bone fragility and fracture risk, is of particular concern because of the major burden of osteoporotic fractures in terms of morbidity and mortality. BMD is not the sole criterion to determine fracture risk. An overall fracture risk assessment that combines multiple risk factors such as the WHO FRAX algorithm provides a more accurate evaluation.

Fracture probability estimated with the FRAX algorithm is, however, underestimated in patients with certain conditions. For example, in the FRAX algorithm, anticancer treatment is not considered as a specific risk factor nor does this algorithm include a “renal” parameter or a measure of sarcopenia and fall risk. Therefore, fracture risk in patients on some anticancer treatments, dialysis, or with severe kidney disease or sarcopenia may be underestimated. Other risk factors such as age and low body mass index (BMI) are included in the FRAX algorithm. However, the algorithm accepts only ages between 40 and 90 years, so not the very elderly and most of the patients with anorexia nervosa (AN), who, in general, are younger than 40 years. Also the management of osteoporosis in the oldest old and in patients with AN is of interest because currently approved therapies for osteoporosis have mainly been investigated in persons until the age of 75 years and are not indicated for use in young individuals.

Reducing fracture risk in frail individuals is of major importance because of the morbidity and mortality associated with fragility fractures. In this consensus paper, we will review the current evidence about the evaluation and management of osteoporosis in frail persons, namely in patients with AN, patients on dialysis, cancer patients, persons with sarcopenia, and the oldest old. Osteoporosis in other frail subsets of the population, such as in HIV positive patients and patients with diabetes mellitus, will not be discussed.

## Osteoporosis in Patients with Anorexia Nervosa

The estimated lifetime prevalence of anorexia nervosa (AN) ranges between 0.6% and 4.2%, but these estimates are likely to be low due to the tendency of some individuals to conceal their illness [1]. AN is 10 times more common in women than men. In this section we will, therefore, discuss only women affected by AN and osteoporosis. The median age of onset of AN is 18 years [1]. The psychological profile of young women with AN is that of women driven to excel, pressurized to fit a specific body image. Osteoporosis is common in AN. About half of these patients suffer from osteoporosis and almost all from osteopenia [2]. Although amenorrhea is not anymore required in the definition of AN, amenorrhea is common and the typical triade consists of low energy intake (generally related to eating disorders), osteoporosis, and amenorrhea.

### Etiology

AN is associated with marked endocrine changes that also impair healthy bone turnover. As mentioned before, amenorrhea is common. In adolescents with AN, menarche is often delayed, contributing to low BMD. However, the severity of bone loss in women with AN is greater than in those with normal-weight hypothalamic amenorrhea, indicating that, in addition to estradiol deficiency, there are other factors including nutritional deficiencies and hormonal abnormalities that contribute to bone loss. GH resistance, low levels of IGF-1, hypercortisolemia, and low levels of testosterone have all been implicated in AN-associated osteopenia and osteoporosis. Abnormalities in hormones regulating appetite (oxytocin, leptin, and peptide tyrosine tyrosine (PYY)) may play an additional role [1]. Therefore, the etiology of bone loss in patients with AN is multifactorial.

### Biological Markers of Bone Turnover

Klibanski et al. observed an uncoupling of bone turnover in adults with AN, with a decrease in markers of bone formation and an increase in markers of bone resorption. However, in adolescents with AN, there is a low bone turnover state with a decrease in the levels of bone formation and resorption, as opposed to an increased bone turnover during normal adolescence [3]. Veronese et al. conducted a meta-analysis of vitamin D status in AN patients [4]. They identified 15 studies (totalizing 927 participants; AN = 408 and healthy controls = 519) and observed that, although AN patients reported similar dietary vitamin D intake compared to healthy controls, AN patients had significantly lower levels of 25OHD and 1,25(OH)_2_D when not using vitamin D supplementation. Conversely, supplementation with cholecalciferol fully normalized serum levels of vitamin D [4].

### Bone Loss and Fracture Risk

Both trabecular and cortical bone sites are affected in AN, but since there is a marked estrogen deficiency component in these women, trabecular osteoporosis (such as at the lumbar spine) is more frequent and prominent [5]. Based on HR-pQCT studies, it has been suggested that bone geometry, microarchitecture, marrow adiposity, as well as bone strength estimates are affected [5]. When AN affects women before reaching their peak bone mass, a reduced bone accrual will impeach them from attaining the optimal BMD, increasing their risk of fracture [6].

Not surprisingly, therefore, a two- to sevenfold increase in fractures has been reported in patients with AN [7, 8]. Rigotti et al. followed a series of 27 women with AN for a median of 25 months and concluded that anorectic women have an increased risk of fracture (RR 7.1; 95% CI 3.2–18.5) and that BMD reductions appear not to be rapidly reversed by recovery from AN [7]. Vestergaard et al., using a case-control Danish Nationwide register study, also observed an increased fracture risk in AN, which persisted more than 10 years after the diagnosis [8].

### Assessment and Management of Osteoporosis in AN

The clinical assessment involves an osteoporosis assessment consisting of BMD measurement and assessment of other risk factors for osteoporosis such as a personal history of low-trauma fractures, a family history of osteoporotic fractures, lifestyle factors, medication, and serum level of 25OHD. In addition, checking for reproductive dysfunction (amenorrhea, follicle-stimulating hormone (FSH), luteinizing hormone (LH), estradiol) and measuring prolactin, thyroid-stimulating hormone (TSH), and human chorionic gonadotropin (hCG) is needed to rule out causes of amenorrhea other than AN.

Table 1 gives an overview of the management of low bone density in patients with AN. AN patients need to be encouraged to gain weight. Recovering a near-normal or normal BMI (18–25 kg/m^2^) will generally help to recover a normal reproductive functioning, which can be assessed by a regular menstrual cycle (not using hormone therapy). Restoration of body weight will also improve BMD although a complete catch-up does not always occur [5]. In addition, AN patients should take adequate calcium (e.g., 1200 mg) and vitamin D (e.g., 800 IU) daily, from diet and supplements.

**Table 1:** Management of low bone density in AN

	Adolescent girls	Postmenopausal women
Lifestyle advise	Decrease exercise and increase weight gain Calcium and vitamin D	Decrease exercise and increase weight gain Calcium and vitamin D
When to initiate pharmacological therapy?		
FRAX	(FRAX is not intended for use in persons < 40 years)	No specific guidelines for adults with AN
DXA	Z-score ≤ −2+ decreasing over time despite all efforts at weight gain [5, 9]	No specific guidelines for adults with AN
Which pharmacological therapy?		
Recommended	Lower-dose physiologic estrogen [10] MHT (100 µg transdermal 17-beta-estradiol with cyclic micronized progesterone)	In adults with osteoporosis MHT (100 µg transdermal 17-beta-estradiol with cyclic micronized progesterone) in women aged 50–60 years* Bisphosphonates* Denosumab* (schemes & doses for postmenopausal osteoporosis)
Not recommended	High-dose estrogen (oral contraception) [11] (“pill” containing 50 µg ethynilestradiol) Androgen replacement [5] Recombinant human insulin-like growth factor 1 (rhIGF-1) (under investigation) [5] Bisphosphonates Denosumab Teriparatide	In adults with AN-associated bone loss Additional research is needed to confirm efficacy and safety of bisphosphonates [12] No data regarding use of Denosumab [5] Role for Teriparatide not yet clear (under investigation) [5, 12]

*Few series

High-dose estrogen–progestin contraception is not an effective treatment option for AN-associated bone loss in adolescents or adults. Indeed, prospective trials have failed to show a benefit of combination estrogen–progestin oral contraceptives in treating AN-associated bone loss in adolescents or adults, although one may be tempted to restore menstruation in these patients using oral contraceptives. Unfortunately, several studies have failed to observe a protective effect of the “pill” containing 50 µg ethynilestradiol [11]. On the other hand, lower-dose physiologic estrogen replacement using MHT such as 100 µg transdermal 17-beta-estradiol with cyclic micronized progesterone in adolescents resulted in BMD gains at the spine and hip compared with placebo, though the therapy did not restore BMD to normal [10]. Although AN patients are also deficient in androgens, androgen replacement is currently not recommended.

Limited data suggest that bisphosphonates may be of benefit in women with AN-associated bone loss. For instance, risedronate for 1 year resulted in a modest gain in BMD compared with placebo [13]. Another study, however, failed to report an improvement using alendronate *vs*. placebo [14]. Furthermore, there are safety concerns with bisphosphonates especially in younger patients who may want to become pregnant. Indeed, animal studies have suggested placental transfer of bisphosphonates and fetal skeletal development involvement, and in a small series of 10 women treated with bisphosphonates during pregnancy, congenital malformations were reported in 20% [15]. Although most other data in literature suggest that preconceptional and first-trimester use of bisphosphonates do not pose substantial fetal risks, it is recommended that, when bisphosphonates are prescribed to females of reproductive age, one should ensure that the patient is and will not become pregnant when using bisphosphonates [15]. Bisphosphonates are generally not approved for this indication, but may be used in elderly with AN. Also denosumab and teriparatide should not be used as first-line therapies in young patients suffering from AN-induced osteoporosis.

## Conclusion

AN predominantly affects women at a young age. As a result, almost all endocrine axes are disturbed. This will lead to osteoporosis in about half of the patients and to osteopenia in almost all of them. The burden due to fractures is important. A multidisciplinary management is needed, involving a gynecologist, endocrinologist, pediatrician (depending of the patients’ age), psychologist, nutritionist, and bone specialist. BMD measurement and an endocrine evaluation are mandatory. Other pathologies associated with weight loss, amenorrhea, and bone loss should be excluded. The ultimate therapy implies body weight restoration and regular cycle recovery. A healthy lifestyle is therefore mandatory. Calcium and vitamin D supplementation is recommended in all patients with AN. MHT is indicated in adolescent girls with sustained low weight, low BMD, and amenorrhea when other causes of amenorrhea have been excluded. Bisphosphonates and denosumab may be useful in adult women with severe bone loss.

## Osteoporosis in Dialysis Patients

### Fracture Risk in Dialysis Patients

Dialysis status is associated with an outstanding risk of fracture compared to the general population. In 2006, results from the international observational cohort DOPPS (Dialysis Outcomes and Practice Patterns Study) were published. Such as in the general population, the risk of hip fracture in the dialysis population was higher in women and increased progressively with age, but, in both genders and in all age categories, the incidence of hip fracture was much higher in dialysis patients. For example, the incidence of fracture in women between 75 and 84 years was 1190 and 3136 per 100,000 patients in the general and dialysis population, respectively [16]. Similar data from USRDS (United States Renal Data System) showed a fourfold higher risk of fracture in dialysis compared to the general population [17]. There is also evidence that these fractures occur earlier in life, and fractures in hemodialysis are associated with higher morbidity and even mortality. A mortality rate as high as 64% in the year following a hip fracture has been described in dialysis patients [18]. Of course, the risk of fracture is also tightly associated with the high risk of falls in this population, which is enhanced by factors such as older age, diabetes, depression, antidepressants, previous falls, malnutrition, and frailty.

### Osteoporosis in Dialysis Patients: Bone Mass Versus Bone Turnover

In clinical practice, bone fragility is evaluated by DXA that measures both cortical and trabecular bone quantity. New CT-scan techniques (quantitative computed tomography (QCT) or high-resolution peripheral QCT (HR-pQCT)) are now available and currently used in clinical research with the main advantage of being able to separate cortical and trabecular areas [19–21]. However, bone quantity is only one piece of the puzzle. Indeed, bone strength, and thus fracture risk, is dependent not only on bone quantity but also on bone quality which is determined by both bone turnover and mineralization, concepts being assessed by bone biopsy and histomorphometry [19, 22].

Bone disorders have been described for more than a century in chronic kidney disease (CKD) patients [22]. In 2006, the international KDIGO (Kidney Disease: Improving Global Outcomes) guidelines proposed a common terminology using the term “Chronic Kidney Disease-Mineral and Bone Disorder” (CKD-MBD) to encompass all possible abnormalities associated with bone disease and mineral disturbances [23]. CKD and still more dialysis status is associated with severe mineral abnormalities, such as hypocalcemia, hyperphosphatemia, and secondary hyper- or hypoparathyrodism. It is beyond the scope of this article to review the pathophysiology of such abnormalities [23, 24] but basically they will be associated with either low (“adynamic bone disease”) or high (“osteitis fibrosa”) bone turnover disease in dialysis patients [22]. The vast majority of basic and clinical research in nephrology is thus focused on turnover abnormalities, and by consequence more on bone quality (turnover) than on bone quantity (osteoporosis) [19]. Briefly, both low and high turnover seems associated with an increase of fracture risk [19–21, 23, 25]. *Sensu stricto*, such a diagnosis requires a bone biopsy but this technique remains relatively invasive, costly, and difficult to interpret and repeat. An important part of clinical research is thus about the role of biomarkers to estimate bone turnover [24]. Currently, the KDIGO recommends the measurement (and monitoring) of both parathyroid hormone (PTH) and bone-specific alkaline phosphatase (b-ALP), even if, as discussed later in this chapter, the interpretation of the results remains frequently difficult and sometimes disappointing [24].

### Diagnosis of Osteoporosis in Dialysis Patients: DXA, FRAX and Biomarkers

Data on the utility of DXA in dialysis patients, notably for predicting the risk of fracture, are scarce and controversial in comparison to the literature in the general population. For this reason, but also because specific therapies for osteoporosis are not routinely used in dialysis, the KDIGO did not recommend routine DXA in dialysis patients when first published in 2009 [23]. This recommendation could, however, change in the next KDIGO recommendations. Indeed, a meta-analysis, including 6 studies and 683 (357 women) dialysis patients, suggested that low BMD was associated with fractures and this was true for all sites, except the femoral neck [26]. However, data were heterogeneous and only cross-sectional. A longitudinal observational study in 485 hemodialysis patients showed that DXA, especially at the hip region, was useful to predict incident fractures but only in patients with low PTH [25]. Beyond fracture risk, some authors suggested that in dialysis patients, osteoporosis and osteopenia were associated with an independent higher risk of mortality [27]. These data could lead to a broader use of DXA in dialysis patients in the future, even if currently the level of evidence is still relatively low. Therefore, at this time, a T-score threshold at which intervention should be started, cannot be recommended. Moreover, there are some specific technologic issues in dialysis patients. Indeed, these patients frequently suffer from spinal osteophytes and high vascular calcifications in the aorta making the interpretation of DXA results at the lumbar region questionable, with a risk of spuriously good results [19, 27]. It is thus suggested to measure BMD in the radius region, especially when there is secondary hyperparathyroidism, and this is another reason why QCT and HR-pQCT are of potential interest in dialysis patients [20].

FRAX does not include any “renal” parameter. This score has not been validated in dialysis patients and is thus considered neither in the KDIGO nor in clinical practice. Therefore, 10-year FRAX risks at which intervention should be initiated cannot be recommended.

If the available literature on bone biomarkers to detect and/or monitor bone turnover abnormalities in dialysis patients is abundant [24], current data on these biomarkers to detect and/or monitor osteoporosis are scarce and controversial. In the general population, biomarkers (especially β C-terminal cross-linked telopeptide (β-CTX) and N-terminal propeptide of type I procollagen (PINP)) may be used to monitor the response to osteoporosis therapy [28]. This is, however, not relevant in dialysis patients as nearly all osteoporotic treatments are not recommended in these patients. The current knowledge on the ability of such biomarkers to detect osteoporosis and/or to monitor bone loss is thus limited and controversial [21, 23]. A recent observational longitudinal study in 81 US dialysis patients measured several biomarkers as well as BMD by DXA and QCT at baseline and after one-year of follow-up [29]. At baseline and after multivariate analysis with age, gender, BMI, and ethnicity as covariates, an association was observed between DXA at the hip and baseline PTH and b-ALP (negative association) and sclerostin (positive association). At the spine, the associations were significant with PTH and sclerostin but also with FGF-23. Considering change in BMD over a one-year period, there was no association between change in BMD at the hip and any biomarker. At the spine, change in BMD was, however, associated with baseline FGF-23 values (negative association) and with change in sclerostin (positive association). The associations observed between change in BMD measured by QCT and biomarkers are also significant but with other biomarkers than those associated with DXA (for example, tartrate-resistant acid phosphatase-5b) and the associations are also different according to the region (hip or spine) [29]. The association between BMD or change in BMD and biomarkers are thus very heterogeneous, making the interpretation of this study difficult. Moreover, each biomarker, including “simple” biomarkers like PTH, has its own analytical and clinical limitations (accumulation in CKD, high intra-individual variation, absence of normal reference values) [24]. Other studies on the same topic are thus urgently needed. For example, for PTH, second- and third-generation PTH assays are currently available, which measure 1–84 and 7–84 PTH (‘intact’ PTH assay) and 1–84 PTH only, respectively. KDIGO experts conclude that both the second- and the third-generation assays are similarly informative. However, the assays have high inter-method variability. Therefore, KDIGO proposes to use a target range for serum PTH based on multiples of the upper limit of the normal values rather than on absolute concentrations [24].

### Therapeutic Strategies in Dialysis Patients

The majority of therapeutic studies in dialysis patients focused on bone turnover abnormalities with, for example, vitamin D or calcimimetics, although the majority of these studies used biomarkers, and not bone biopsies, as endpoint [19]. Clinical studies reporting fractures as hard clinical endpoint are even more scarce. One large RCT suggests that fractures could be reduced in dialysis patients with hyperparathyroidism when treated with calcimimetics [30]. This was, however, a post hoc analysis with statistical adjustments, while the primary study was negative for the fracture endpoint [31].

There are few studies about specific osteoporosis therapies in dialysis patients as dialysis status was an exclusion criterion in all large interventional trials on this topic [19–21]. The role of bisphosphonates in dialysis patients is still a subject of debate. Data in literature are scarce in severe CKD non-dialysis patients and even more limited in dialysis patients [20]. Some publications, however, suggest positive results on BMD [32]. Because 50% of the administered dose of bisphosphonates gets excreted by the kidneys, the dosage used in these studies is lower than in the general population. Limitations of these studies are the design, sample size, and follow-up (6–12 months). In theory, bisphosphonates are contraindicated in patients with low bone turnover. For this reason, the KDIGO recommends to perform a bone biopsy before a potential treatment with bisphosphonates. Also raloxifene has been hardly studied in dialysis patients. The safety profile seems acceptable in postmenopausal women on hemodialysis, but long-term data are lacking. The beneficial impact on BMD has been suggested by some prospective and sometimes randomized studies but the sample sizes and follow-up times were always limited (at best 26 patients in the treated group and follow-up of 1 year) [33]. Likewise, there are few clinical data on the benefit of strontium ranelate in dialysis patients and some authors have underlined the risk of osteomalacia with this treatment in dialysis patients [34]. Recent osteoporosis therapies have theoretical interest in dialysis patients. Denosumab has been shown to be effective in dialysis patients but the numbers of included patients were low and all studies were open-label and non-randomized. Moreover, dialysis patients are at higher risk of developing severe hypocalcemia with denosumab [35]. According to some authors, this antiresorptive therapy should also be avoided in dialysis patients with low bone turnover [20]. Teriparatide and other PTH analogs are theoretically interesting, especially in dialysis patients with low bone turnover but, once again, available data are limited to case reports and pilot studies [36]. Lastly, also in the context of low bone turnover disease, new anti-sclerostin antibodies could be beneficial but until now, only animal studies are available [37].

### Future Research

Because of the high risk of fracture in dialysis patients, there is a clear interest to promote clinical research in the field of bone disease. Nephrologists should certainly move from an “only bone turnover” (bone quality) point of view to a broader vision including the concept of bone quantity. This change of concept is necessary because many new and potentially useful therapies are now available. However, there is still need to clarify the place of DXA, QCT, and biomarkers to predict the risk of fractures and/or to evaluate or monitor BMD.

Cardiovascular mortality is the first cause of death in dialysis patients. At least in part, vascular calcifications explain this cardiovascular over-mortality. The pathophysiology of these vascular calcifications has been suggested to share similar pathways with bone physiology. In the future, potential connections between bone (and osteoporosis) and vessels (and vascular calcifications) will be an interesting field of basic and clinical research. Some data suggest an inverse correlation between vascular calcifications and osteoporosis [20, 27]. Therefore, future studies with osteoporosis therapies should be designed with two endpoints; the effect on bone (BMD, biomarkers and ideally fracture) and the effect on vessels (vascular calcification and ideally cardiovascular mortality).

## Conclusion

Dialysis status is associated with a major risk of fractures compared to the general population. Furthermore, fractures in dialysis patients occur earlier in life and are associated with a higher morbidity and mortality. The assessment of bone health and fracture risk in dialysis patients may be difficult because of the heterogeneous association between (change in) BMD and biomarkers. Moreover, the FRAX algorithm does not include dialysis status; DXA is, until now, not recommended by KDIGO and bone biopsies remain relatively invasive and difficult to interpret. Since dialysis status was an exclusion criterion in all large RCTs, the role of bisphosphonates is still a subject of debate. Denosumab has been shown to be effective but with a risk of developing severe hypocalcemia. Teriparatide and other PTH analogs may be interesting in dialysis patients with low bone turnover, in whom antiresorptives should be avoided.

## Osteoporosis in Cancer Patients

The term “bone health” in cancer patients encompasses the impact of metastatic bone disease and the effects of cancer therapy on bone mass and fracture rate. Metastatic bone disease is most commonly seen with specific cancer types, notably those arising from the breast, prostate, lung, and kidney, as well as multiple myeloma. Bone metastases weaken the structural integrity of bone, putting patients at increased risk of bone complications, skeletal-related events (SREs), including pathologic fracture, spinal cord compression, or subsequent radiation or surgery to the bone. SREs are associated with increased morbidity and mortality, decreased quality of life, and increased treatment costs. Many patients with bone metastasis also experience bone pain that can be severe and debilitating. Antiresorptive agents markedly reduce the incidence of SREs and delay the occurrence of severe bone pain. The reader is referred to a recent review for this topic [38]. A second connection between cancer and bone is that most drugs used to treat hormone-responsive tumors have a deleterious indirect effect on bone turnover, BMD, and bone quality. In the elderly, cancer treatment-induced bone loss (CTIBL) is superimposed on physiological bone loss. This chapter focuses on CTIBL in breast cancer and prostate cancer in the adjuvant setting. There has been less interest for bone health in patients cured from other tumors, although bone loss after stem cell transplantation at last received more attention.

### Breast Cancer

Aromatase inhibitors (AIs) increase overall survival in RCTs against tamoxifen and have become the first-line hormonotherapy in the adjuvant setting of breast cancer. Bone loss is their main side effect. AI therapy is associated with an average 2% loss of lumbar spine BMD per year, and the effects of AIs on cortical bone and bone strength appear to be largely underestimated by classical dual-energy X-ray absorptiometry (DXA) [39]. Risk of fracture is 2–4 times higher in women treated with adjuvant AIs than with tamoxifen or placebo [38, 39]. The increased risk is independent of the type of AI and, with the exception of ABCSG-18, the only trial where fracture incidence was the primary endpoint, the risk has been underestimated because fractures were only reported as adverse events in oncology trials. The absolute risk of fracture in women treated with AIs ranges from 1–18%. Data from ABCSG-18 show a fracture rate of 9.6% after 3 years and 26% after 7 years in the placebo group on AIs only [40].

Published guidelines recommend that women with breast cancer receiving an AI or ovarian suppression therapy have their bone health monitored for fracture risk, for example, with BMD measurement and the FRAX algorithm [38]. In FRAX, however, anticancer treatments are not included as a unique risk factor and enter in the “secondary osteoporosis” group, which underestimates the effect of these therapies on fracture risk [38]. As for other conditions characterized by an increased fracture rate, patients receiving AIs should be advised to consume a calcium-enriched diet and/or receive calcium supplements, exercise moderately (resistance and weight-bearing exercise), and take 1000–2000 international units (IU) of vitamin D everyday. Table 2 summarizes treatment guidelines and possible therapeutic schemes with antiresorptives for women on AI. Guidance from expert groups for premenopausal women with therapy-induced early menopause recommends the use of antiresorptives if the BMD Z-score is <2.0 [41]. In postmenopausal women, the consensus from expert panels recommends treatment with antiresorptives in patients receiving AI therapy with a T-score <2.0 or with two or more clinical risk factors for fracture [38].

**Table 2:** Antiresorptives in women with early breast cancer

	Prevention of CTIBL in women with early breast cancer treated with aromatase inhibitors		Prevention of metastases and improving diseases outcomes in women with early breast cancer	
Who?	When? (according to different expert groups)	Which agents are recommended?	Which agents?	Level of evidence and grade of recommendation^a^
Premenopausal women receiving adjuvant ovarian suppression	Z-score <2.0 [41] T-score <1.0 [42] 10-year fracture risk (FRAX) [43] ≥3% for hip fracture ≥20% for major fracture	Zoledronic acid 4 mg i.v. q6mo [44] Other BP do not have a sustained effect on BMD [44]	Clodronate 1600 mg daily [38, 44] Zoledronic acid 4 mg i.v. q6mo [38, 44] Zoledronic acid 4 mg i.v. q3-4w for 6 doses, then q3mo for 8 doses, then q6mo for 5 doses [45]	BP reduce the frequency of bone metastases and improve breast cancer survival: **I, A** [38, 44]
Postmenopausal women	T-score < −2.0 or ≥ 2 clinical risk factors for fractures^b^ [38, 39, 42, 44, 46] I, A [39] 10-year fracture risk (FRAX) [43] ≥3% for hip fracture ≥20% for major fracture	BP prevent bone loss: **I, A** [44] Alendronate 70 mg p.o. weekly [38, 42, 44, 46] Risedronate 35 mg p.o. weekly [38, 39, 42, 44] Clodronate 1600 mg p.o. daily [39, 44] Ibandronate 150 mg p.o. monthly [38, 39, 42, 44] Zoledronic acid 4 mg i.v. q6mo [38, 39, 42, 44] Pamidronate 90 mg i.v. q3mo [38] Denosumab prevents bone loss **(I, A)** and fractures [40] Denosumab 60 mg s.c. q6mo [38–40, 44]	In women at intermediate or high risk of recurrence Clodronate 1600 mg daily [38, 44] Zoledronic acid 4 mg i.v. q6mo [38, 44] Zoledronic acid 4 mg i.v. q3-4w for 6 doses, then q3mo for 8 doses, then q6mo for 5 doses [45]	BP reduce the frequency of bone metastases and improve breast cancer survival: **I, A** [38, 44, 47]
	Guidelines for AI-induced bone loss are currently reviewed in the light of the anti-tumoral effects of antiresorptive treatment (see right side of table) [46]		Recommended by expert groups (joint effort of various societies, including IOF [44])	
			Data on Denosumab pending – Denosumab 60 mg s.c. q6mo improved disease-free survival in high-risk patients (ABCSG-18 [48])	

^a^Marked in bold: level of evidence (I-V), grade of recommendation (A-E)

^b^Clinical risk factors for fracture include: age > 65 years, T-score <1.5, smoking (current or history of), BMI < 24 kg/m², family history of hip fracture, personal history of fragility fracture above age 50, oral glucocorticoid use for >6 months [38, 39]

Data from randomized clinical trials (RCTs) in >5000 patients show that bisphosphonates and denosumab administered at doses and schedules that are most often similar to those used for postmenopausal osteoporosis can prevent bone loss in women with breast cancer and even lead to an increase in BMD [38, 39]. The ABCSG-18 trial, which randomized postmenopausal women on AIs to denosumab 60 mg q6m or placebo, found that active treatment reduced the risk of first clinical fracture (the primary endpoint) relative to placebo by 50%. Five years following randomization, 15% of placebo patients but little over 5% of denosumab-treated patients had experienced a fracture. A significant protective effect was seen both in women with a baseline T-score < -1 and in those with a T-score ≥ −1 [40]. These new findings will have to be considered when updating guidelines for the prevention of AI-induced bone loss, especially given that denosumab was not associated with additional toxicity. In particular, there was no concern over osteonecrosis of the jaw or atypical femoral fractures [40]. Important additional evidence for the use of antiresorptives in the adjuvant setting is provided by the recent Early Breast Cancer Trialists’ Collaborative Group (EBCTCG) meta-analysis of data from postmenopausal breast cancer patients showing that adjuvant zoledronic acid and clodronate could reduce recurrence rate and prolong survival. Compelling evidence from this meta-analysis of trial data of >18,000 patients supports clinically significant benefits of bisphosphonates on the development of bone metastases and breast cancer mortality in postmenopausal women or those receiving ovarian suppression therapy [47]. An international panel of experts has recently recommended that bisphosphonates, either iv zoledronic acid or oral clodronate, are considered as part of the adjuvant breast cancer treatment in this population. Data from the adjuvant use of denosumab in this setting are eagerly awaited [44, 48].

### Prostate Cancer

Androgen deprivation therapy (ADT) is the cornerstone of treatment in prostate cancer but has several adverse effects, especially on bone health. ADT can be achieved by castration, luteinizing hormone-releasing hormone (LHRH) agonists or antagonists. Men undergoing ADT should have their bone health monitored for fracture risk [49]. As for breast cancer, FRAX underestimates the effects of hormone therapy on fracture risk. ADT leads to accelerated bone loss and an increase in fracture rate, as evidenced by large retrospective epidemiological studies [50, 51]. Men treated with LHRH agonists lose 1–5% of BMD within the first year and fracture risk increases with treatment duration [50]. A matched-cohort study of almost 20,000 men found that the risk of fragility fracture (all sites) was 17.2% for those on ADT (mean duration 6.5 years) compared with 12.7% among men not on ADT (HR 1.65; 95% CI 1.39–1.54) [51]. Increasing age is an independent risk factor for fractures, and fracture in prostate cancer more than doubles mortality. The recent introduction of the androgen synthesis inhibitor abiraterone for castration-resistant prostate cancer (CRPC) could aggravate the problem because of its impact on endogenous cortisone production. Abiraterone must indeed be combined with 10 mg prednisone and ADT has to be continued anyway. Abiraterone and other new agents significantly extend overall survival in CRPC, so that prolonged exposure to steroids and ADT may be expected, potentially increasing the risk of osteoporotic fracture. Because of its favorable toxicity profile, abiraterone is especially suited to elderly who are not good candidates for chemotherapy [52].

Table 3 summarizes possible therapeutic schemes with antiresorptives for men on ADT. Alendronate, risedronate, zoledronic acid, and pamidronate have all been shown to prevent BMD loss in patients with locally advanced prostate cancer under ADT [38]. 6–12 monthly zoledronic acid and 6-monthly denosumab are most often used. Prostate cancer is essentially a disease of elderly men who are more likely than younger men to require dose adjustment for renal impairment, which makes denosumab more attractive than bisphosphonates. Moreover, only denosumab has a specific license for ADT-induced bone loss. In a placebo-controlled trial of denosumab in 1468 men receiving ADT for non-metastatic prostate cancer, 3 years of denosumab treatment at doses used for the treatment of osteoporosis led to a 62% relative reduction in new vertebral fractures [53]. Although antiresorptive therapies are especially important for elderly patients with cancer, they are typically underutilized in this population and guidelines for this indication have received less attention. They should probably be broadly similar to those in breast cancer and recent guidelines advocate that all men aged over 75 years under ADT should receive antiresorptive agents at doses used to prevent osteoporosis [46]. Underuse of antiresorptive therapies may be more detrimental in elderly compared with younger patients because of multiple fracture risk factors, including physiological decreases in BMD and increases in fracture rate with advancing age.

**Table 3:** Antiresorptives in men with prostate cancer

Prevention of CTIBL in men with prostate cancer treated with androgen deprivation therapy		Prevention of metastases and improving diseases outcomes in men with prostate cancer	
When? (according to different expert groups)	Which agents are recommended?	Which agents?	Level of evidence and grade of recommendation^a^
T-score < −2.0 or ≥2 clinical risk factors for fractures^b^[38] T-score ≤ −2.5 [46, 54] 10-year fracture risk (FRAX) [43, 46] ≥3% for hip fracture ≥20% for major fracture	BP prevent bone loss: **I, B** [38], but various treatment schemes Alendronate 70 mg p.o. weekly [38] Risedronate 35 mg p.o. weekly [38] Zoledronic acid 4 mg i.v. q3mo [55] Zoledronic acid 4 mg i.v. q6mo [38] Zoledronic acid 5 mg i.v. q12mo [43] Pamidronate 90 mg i.v. q3mo [38] Denosumab prevents bone loss & vertebral fractures: **I, B** [38] Denosumab 60 mg s.c. q6mo [38, 53]	Denosumab 120 mg s.c. monthly [38]	Denosumab delays bone metastasis in castrate-resistant prostate cancer, but no effect on overall survival: **I, B** [38]

^a^Marked in bold: Level of evidence (I-V) - Grade of recommendation (A-E)

^b^Clinical risk factors include: age >65 years, T-score <1.5, smoking (current or history of), BMI < 24 kg/m², family history of hip fracture, personal history of fragility fracture above age 50, oral glucocorticoid use for >6 months [38]

### Hematopoietic Stem Cell Transplantation

Hematopoietic stem cell transplantation (HSCT) is the treatment of choice for many patients with malignant and non-malignant hematological diseases. Osteoporotic fractures constitute one of the main long-term complications. The etiology of bone loss after HSCT is multifactorial, including the use of corticosteroids and other immunosuppressive drugs, release of inflammatory cytokines, and treatment-induced hypogonadism. Even if patients undergoing HSCT are most often less than 50 years, the incidence of osteopenia and osteoporosis in adults after HSCT approaches 50% after 5 years and 20% after 2 years, respectively. The degree of bone loss is most often severe, especially after allo-HSCT, and cortical bone appears to be affected more than trabecular bone [56]. The relative risk of fractures is increased seven- to ninefold compared with 45- to 64-year-old adults in population-based cohort studies [57].

All HSCT patients should receive dietary and lifestyle advice, including calcium and vitamin D supplementation. Menopause hormone therapy (MHT) should be considered in menopausal women, and testosterone potentially in hypogonadal men. Antiresorptives used in this population are mostly bisphosphonates but there are few randomized trials [58]. The pamidronate trials included the largest number of patients and appear to be more representative of the real effects of bisphosphonates in this population. A randomized study including 99 patients under HSCT with or without five 60-mg pamidronate infusions over 1 year showed that in the pamidronate group, BMD remained stable at lumbar spine but still decreased at the femoral neck and total hip [59]. In a smaller study, using higher doses of pamidronate, comparable to the doses used for the treatment of bone metastases, similar findings were reported [60]. There is no reported experience with denosumab in patients after HSCT.

## Conclusion

To conclude, AIs, ADT, and HSCT are associated with bone loss and fracture risk. Therefore, individuals on these therapies should have their bone health monitored for fracture risk. The FRAX algorithm, however, does not consider anticancer treatment as a specific risk factor and thus underestimates the effect of these therapies. Cancer patients treated with AIs, ADT, and HSCT should receive dietary and lifestyle advice, including adequate calcium and vitamin D intake. Furthermore, in patients on AIs and ADT, bisphosphonates and denosumab administered at doses similar to the treatment of postmenopausal osteoporosis can prevent bone loss. Denosumab has also been shown to reduce fracture risk in both conditions. Moreover, antiresorptives in the adjuvant setting reduce recurrence rate in postmenopausal women with breast cancer. The few RCTs with bisphosphonates in adults with HSCT showed reduced bone loss, while MHT may be considered in menopausal women and testosterone in hypogonadal men.

## Osteoporosis in Sarcopenic Patients

Sarcopenia corresponds to a progressive and generalized loss of muscle mass combined with either loss of muscle strength or physical performance [61]. Consequences of sarcopenia include physical disability, nursing home admissions, depression, hospitalizations, and mortality, thus an increased morbidity and mortality essentially similar to the consequences of osteoporosis.

During the last decade, bone and muscle were increasingly recognized as interacting tissues, not only because of their adjacent surfaces or as a result of the mechanical effects of muscle loading on bone [62]. The “bone-muscle-unit” is now widely considered as the site of privileged exchanges in which the two tissues communicate via paracrine and endocrine signals to coordinate their development and adapt their response to loading and injury from embryologic stages to involution [63, 64]. During growth, muscle area seems closely correlated with bone parameters such as bone mineral content and femoral circumference. Growing evidence suggest that sarcopenia and osteoporosis share many common pathways including the sensitivity to reduced anabolic hormone secretion, increased inflammatory cytokine activity, anabolic or catabolic molecules released by the skeletal muscle or bone cells (i.e., myokines and osteokines), and eventually reduced physical activity [64, 65].

The concept bone-muscle-unit is evidenced phenotypically by the observation of a linear relationship between BMD and lean body mass at various ages [66]. The muscle–bone cross-talk is also supported by preclinical data, showing its presence even before birth in mammals [63]. The muscle secretome consists of several hundred secreted peptides, providing a whole new paradigm for understanding how muscles communicate with other organs, including bones [63]. Several molecules released by muscle affect bone, including IGF-1 (insulin-like growth factor-1), fibroblast-growth factor-2, interleukin (IL)-6, IL-15, myostatin, osteoglycin, FAM5C (family with sequence similarity 5, member C), Tmem119 (transmembrane protein 119), irisin, and osteoactivin [63]. However, much less studies were dedicated to studying the reverse channel (i.e., from bone to muscle). Both osteoblasts and osteocytes were shown to secrete cytokines. The effects on muscle of prostaglandin E2 and Wnt3A (wingless-type MMTV integration site family, member 3A) which are secreted by osteocytes, osteocalcin, and IGF-1, produced by osteoblasts, and sclerostin secreted by both cell types, are however well documented [63].

With adipose tissue also involved in the complex bone–muscle interaction came the suggestion that obesity, sarcopenia, and osteoporosis could be concomitantly found in a subset of the population, presenting with an entity called osteosarcopenic obesity [67]. The mechanism underlying this condition is an increase in total and/or abdominal adipose tissue that causes an increase in pro-inflammatory cytokines as well as some hormonal disturbances leading to losses of both muscle and bone. The decrease in muscle and bone is associated with a decrease in physical activity leading to a vicious cycle of progressive loss of muscle and bone and a gain in fat [67]. It is likely that these individuals will present with poorer clinical outcomes caused by the cascade of metabolic abnormalities associated with the changes in their body composition. This view was supported by the observation that obese subjects with low muscle mass (sarcopenia) or strength (dynapenia) have an increased risk of osteoporosis and non-vertebral fracture relative to obese alone counterparts. These findings imply that sarcopenic and dynapenic obese individuals require close monitoring of bone health during ageing.

### Bone–muscle Interaction in Clinical Trials

Several studies support the in vivo association of low BMD and sarcopenia. In 679 men aged 40–79 years from the European Male Ageing Study, sarcopenia was associated with low BMD and osteoporosis [68]. Similarly, in 17,891 subjects from various ethnicities, each standard deviation increase in relative appendicular skeletal muscle mass resulted in a 37% reduction in the risk of osteopenia/osteoporosis and subjects with sarcopenia were 2 times more likely to have osteopenia/osteoporosis [69].

In a study in 591 inpatients, sarcopenia was present in 64% of women and 95% of men who recently experienced a hip fracture [70]. Moreover, sarcopenia was associated with lower ability to perform activities of daily living compared to presarcopenia. This may reflect an increased risk of post-hip fracture complications, additional health resources utilization, and higher incidence of contralateral hip fracture. In a population of young patients (20–69 years) with a femoral neck fracture, those with low-energy trauma have significantly lower femoral neck BMD and fat-free mass than those with other trauma mechanisms [71]. These results re-emphasize the association between low bone and muscle mass in patients with hip fractures, and the need for a comprehensive management of these patients.

Furthermore, in Chinese community-dwelling elderly aged 65 and older, sarcopenia was a predictor of fracture risk independent of BMD and other clinical risk factors, and the diagnosis of sarcopenia added incremental value to the FRAX algorithm in predicting incident fracture risk [72].

Finally, in a cross-sectional study in 680 men and women with a mean age of 79 years, sarco-osteoporotic individuals were more likely to have a lower MNA (mini-nutritional assessment) and BMI compared to normal elderly [73]. In Italian hip fracture patients, a low intake of calories, protein, and leucine was associated with reduced muscle mass [74]. This illustrates the importance of detecting nutritional deficits and optimizing nutritional status in patients with osteoporosis and sarcopenia in order to prevent poor clinical outcomes such as falls and fractures.

### Interventions Targeting both Muscle and Bone

At this stage, no RCTs assessing the concomitant effects of a new chemical entity (NCE) on bone and muscle have been published. This may be, in part, related to the absence of guidelines supporting regulatory studies for NCE to manage sarcopenia [75]. However, a better understanding of the interconnection of bone and muscle may shift our treatment paradigm to “kill two birds with one stone,” i.e., to treat sarcopenic patients and prevent fractures, as suggested by Gingis et al. [64].

A potential strategy is to target pathways that centrally regulate both bone and muscle (e.g., growth hormone (GH) and GH secretagogues, androgens, selective androgen receptor modulators, and vitamin D) or to investigate newly emerging pathways that might facilitate the communication between the two tissues (e.g., activin signaling inhibitors, including myostatin-neutralizing antibodies/propeptides, recombinant follistatin, follistatin derivatives, and soluble activin receptors or myokines) [63, 64]. An overview of the results of human clinical studies with these agents has recently been published [64].

All authors acknowledge the critical importance of regular exercise and adequate nutrition to optimize peak bone mass and maintain bone and muscle health throughout live [63, 67]. In this perspective, two intervention trials are worth a comment. Bauer et al. reported, in a well-designed 13-week RCT, the beneficial effects of a vitamin D- and leucine-enriched whey protein oral supplement on muscle mass and lower-extremity function among sarcopenic elderly [76]. Knowing the importance of vitamin D and proteins for bone health, this nutritional supplement could also take place in the armamentarium against osteoporosis [77, 78]. Chahal et al. investigated the impact of various intensities and frequencies of loading doses of physical activity on knee extension torque and broadband ultrasound attenuation at the heel in middle-aged women. They concluded that physical activity, especially at high intensity level and high frequency range, may have beneficial effects on muscle strength and bone density in this population [79].

## Conclusion

Osteoporosis and sarcopenia are two disorders predominantly affecting elderly patients and responsible for a major clinical and financial burden. Increase in life expectancy in most countries and in both sexes makes their diagnosis, prevention, and treatment a major social and ethical, yet unmet, medical need. Genetic, developmental, paracrine, endocrine, and lifestyle factors have dual effects on bone mass and muscle mass and function.

The evidence of biochemical and molecular interactions between the two tissues needs to be further explored for the development of NCE against these twin conditions of aging. Targeting pathways that centrally regulate bone and muscle or newly pathways that facilitate communication between the two tissues are the directions for the identification of NCE, which could simultaneously prevent, reduce, or restore bone and muscle wasting. It seems wise for companies developing such agents to include, within the secondary endpoints of their trials, outcomes parameters (e.g., DXA, biochemical markers, imaging, quality of life) reflecting the effect of these drugs on bone and muscle. However, the importance of physical exercise and the need for a balanced diet providing sufficient amounts of proteins, calcium, vitamin D, and various micronutrients should not be underestimated.

## Osteoporosis in the Oldest Old

### Osteoporosis and Osteoporotic Fractures in Old Age: A Challenge

The incidence of osteoporotic fractures increases with age. Today, the cumulative incidence of hip fractures in women at the age of 80 is close to 30% [80]. Vertebral fractures are even more common, with a prevalence of more than 40% in women older than 80 [81]. Moreover, the burden of osteoporosis will only increase in the future because of the aging of the population. In Belgium, it is expected that the number of osteoporotic fractures will increase with 25% in the next 10 years. Osteoporosis in old age is a challenge because of its significant burden in terms of morbidity, mortality, and economic cost.

### Elderly Persons with Osteoporosis are Frail Elderly

According to the World Report on Ageing and Health of the World Health Organization (WHO) [82], *healthy aging* is the process of developing and maintaining the *functional ability* that enables well-being in older age. Functional ability is made up of the *intrinsic capacity* of the individual (the composite of all the physical and mental capacities of the individual), relevant *environmental characteristics*, and the interactions between the individual and the environment. An individual may have reserves of functional ability that he or she is not drawing on. These reserves contribute to the resilience of the individual. *Resilience* is the ability to maintain or improve a level of functional ability in the face of adverse events. *Frailty* can be considered as the progressive age-related decline in physiological systems that results in decreased reserves of intrinsic capacity, which leads to extreme vulnerability to stressors and increases the risk of adverse health outcomes [83].

Elderly patients with osteoporotic fractures are not “average” elderly, but should be considered as frail persons, with a high prevalence of underlying comorbidities and at risk of functional deficits [84]. Indeed, in old age, osteoporosis and osteoporotic fractures tend to occur in a particularly frail subset of the population [85]. This frailty will be reflected in poor post-fracture outcomes, such as functional decline, loss of quality of life, and an increased mortality which continues to be observed more than 10 years after the fracture [86].

### Under-diagnosis and Under-Treatment of Osteoporosis in Old Age

Despite the increasing evidence for the frequency and severity of osteoporosis in the elderly, osteoporosis continues to be under-diagnosed and under-treated, particularly in individuals over the age of 80. This may, at least partly, be explained by the fact that evidence of the anti-fracture efficacy of osteoporosis treatment comes mainly from RCTs in women with a mean age of 70 to 75 years. Thus, there is an urgent need for treatment options with documented efficacy in older individuals, not only against vertebral fractures but even more so against non-vertebral fractures, as these account for most of the morbidity and mortality associated with osteoporosis. Treatment options should also be proven to be safe in elderly who are frail, with comorbidities and at increased risk of adverse events.

### Treatment of Osteoporosis in the Oldest Old

In this chapter, the evidence about the efficacy and safety of the available osteoporosis therapies in the elderly, and especially the oldest old (≥80 year), is discussed. Non-pharmacological interventions such as fall prevention strategies play an essential role in the treatment of osteoporosis, also in elderly, but will not be discussed.

#### Calcium and Vitamin D Supplementation in old Age

One of the main determinants of bone loss in old age is calcium and vitamin D deficiency and that is why combined calcium and vitamin D supplementation has become one of the main components to reduce bone loss and fracture risk in old age. Low levels of 25-hydroxyvitamin D (25OHD) occur in all age groups; 2–30% of adults in European countries have a serum 25OHD level below 10 ng/ml, but this may rise to more than 80% in institutionalized elderly [87]. In fact, a gradual decline of 25OHD is observed from healthy adults over independent elderly to institutionalized persons and hip fracture patients [87]. Despite the observation that the absorption of vitamin D_3_ and its metabolism into 25OHD and 1,25-dihydroxyvitamin D (1,25(OH)_2_D) is well preserved in elderly without liver or kidney disease, elderly are at risk of hypovitaminosis D because of low vitamin D intake and decreased capacity of the skin to produce vitamin D_3_ together with less sun exposure [87]. Therefore, elderly and especially those in institutions have lower levels of 25OHD compared to young individuals. Hypovitaminosis D lowers the intestinal calcium absorption and induces a negative calcium balance, which may be enhanced by insufficient calcium intake. This stimulates the secretion of PTH, which enhances bone turnover, induces osteoporosis and increases fracture risk. Low vitamin D may also increase fracture risk by increasing the risk of falling apparently through an effect on balance and muscle strength [78].

Adequate vitamin D status is therefore essential in the prevention of bone loss and osteoporotic fractures. A daily intake of 800 IU of vitamin D is recommended for individuals aged ≥ 71 years in order to achieve a serum 25OHD level of at least 20 ng/ml as this meets the requirements of at least 97.5% of the population [88]. One of the reasons why individual RCTs and meta-analyses failed to show a reduction in fracture risk with calcium and vitamin D may be the lack of targeting of supplementation to persons at risk of a negative calcium balance and/or vitamin D deficiency, such as individuals aged ≥ 75 years and institutionalized elderly. This is illustrated by a recent meta-analysis that found that vitamin D with calcium reduced the risk of hip fractures in institutionalized but not in community-dwelling elderly as the latter group is less likely to have calcium and/or vitamin D deficiency [89].

Thus, combined supplementation with calcium and vitamin D is an essential component to reduce bone loss and fracture risk in the elderly. However, osteoporosis treatment, on top of calcium and vitamin D, should be considered in older individuals with osteoporosis and osteoporotic fractures.

#### Pharmacological Osteoporosis Treatment in Old Age

Table 4 gives an overview of the anti-fracture evidence of currently approved pharmacological osteoporosis medication in old age.

**Table 4:** Relative risk (95% CI) of new vertebral, hip and non-vertebral fractures compared with placebo in very elderly women receiving currently available osteoporosis treatments

	RCT	Included participants	N	Mean age (years)	Vertebral fractures	Hip fractures	Non-vertebral fractures
Alendronate	Post hoc analysis FIT Vertebral Fracture Arm (3 years) [90]	Women aged 75–82 years	539	Not specified	**RR 0.62** **95% CI 0.41–0.94** ***p*** < **0.05** *p*_interact < 75 & ≥ 75 years_ NS	–	–
	Pooled analysis FIT Vertebral and Clinical Fracture Arm with low BMD (3–4 years) [91]	Women aged 55–80 years 55- < 65 years 65- < 70 years 70- < 75 years 75–85 years	3658		**RR 0.55** **95% CI 0.37–0.83** (constant RR)	**RR 0.47** **95% CI 0.27–0.81** (constant RR)	–
	Axelsson et al. [92] (5 years)	Women aged 71.1–92.3 years with a prior fracture	110,190	82.4 Years	–	**HR 0.72** **95% CI 0.61–0.85** ***p*** < **0.001**	–
Risedronate	HIP - arm 2 (3 years) [93]	Women aged ≥ 80 years with at least one non-skeletal risk factor for hip fracture or T-score at FN ≤4 or ≤3 + hip axis length of ≥ 11.1 cm	3886	83 Years	–	RR = 0.8 95% CI 0.6–1.2 *p* = 0.35	10.8% (Risedronate) versus 11.9% (placebo); *p* = 0.43
	Post hoc pooled analysis VERT-NA, VERT-MN and HIP (3 years) [94]	Women aged ≥ 80 y with T-score ≤2.5 at FN or at least one prevalent vertebral fracture	1392	83 years	**HR 0.56** **95% CI 0.39–0.81** ***p*** = **0.003** p _interact < 80 & ≥ 85 years_ NS	–	14.0% (Risedronate) versus 16.2% (placebo) *p* = 0.66
Zoledronic acid	Post hoc analysis HORIZON-PFT and RFT (3 years) [95]	Women aged ≥ 75 years with T-score ≤ −2.5 at FN or ≥ 1 vertebral or hip fracture	3888	79.4 years	**HR 0.34** **95% CI 0.21–0.55** ***p*** < **0.001** *p*_interact < 75 & ≥ 75 years_ NS	HR 0.82 95% CI 0.56–1.2 *p* = 0.297 ***p***_**interact** < **70 &** ≥ **75 years**_ **SS**	**HR 0.73** **95% CI 0.60–0.90** ***p*** = **0.002** *p*_interact < 70 & ≥ 75 years_ NS
Denosumab	Post hoc analysis FREEDOM (3 years) [96]	Women aged ≥ 75 years	2471	78.2 years	–	**0.9% (denosumab)** ***versus*** **2.3% (placebo)** ***p*** < **0.01; ARR 1.4%** *p*_interact < 75 & ≥ 75 years_ NS	–
	Preplanned analysis FREEDOM (3 years) [97]	Women aged ≥ 75 years	2471	78.2	**RR 0.36** **95% CI 0.25–0.53** *p*_interact < 75 & ≥ 75 years_ NS	–	RR 0.84 95% CI 0.63–1.12 p _interact < 75 & ≥ 75 years_ NS
Strontium ranelate	Preplanned pooled analysis SOTI and TROPOS (3 years) [98]	Women aged 80–100 years	1488	83.5 years	**RR 0.68** **95% CI 0.50–0.92** ***p*** = **0.013**	RR 0.68 95% CI 0.42–1.10 *p* = 0.112 (not powered)	**RR 0.69** **95% CI 0.52–0.92** ***p*** = **0.011**
	Preplanned pooled analysis SOTI and TROPOS (5 years) [99]	Women aged 80–100 years	1489	83.5 years	**RR 0.69** **95% CI 0.52–0.92**	RR 0.76 95% CI 0.50–1.15 (not powered)	**RR 0.73** **95% CI 0.57–0.95**
Teriparatide	Prespecified subgroup analysis FPT (19 months) [100]	Women aged ≥ 75 years	244	78.3 years	**RR 0.35** ***p*** < **0.05** p _interact < 75 & ≥ 75 years_ NS	–	RR 0.75; *p* = 0.661 (not powered) p _interact < 75 & ≥ 75 years_ NS

Results in bold indicate significant results

*FN* femoral neck, *LS* lumbar spine, *y* years, *ITT* intention to treat, *NS* not significant

##### Alendronate

The efficacy of alendronate as an antiresorptive agent was established by the Fracture Intervention Trial (FIT) in postmenopausal women with a prevalent vertebral fracture as well as in postmenopausal women with a T-score ≤ −1.6 at the femoral neck (mean age 70.8 and 67.7 years, respectively) [101, 102].

A post hoc analysis of the FIT-trial has evaluated the anti-fracture efficacy of alendronate in postmenopausal women with the highest fracture risk. This analysis included a subgroup with patients aged ≥75 years (range 75–82 years) [90]. After 3 years, alendronate significantly reduced the risk of vertebral fracture by 38% (RR 0.62; 95% CI 0.41–0.94) in women aged ≥75 years compared to 51% in the younger age group (RR 0.49; 95% CI 0.35–0.68). This study was followed by another analysis based on pooled data from both FIT arms [91]. Focus of this analysis was to calculate age-specific fracture rates (55 to <65 years, 65 to <70 years, 70 to <75 years, and 75–85 years). Relative risk reductions for hip (RR 0.47; 95% CI 0.27–0.81) and vertebral (RR 0.55; 95% CI 0.37–0.83) fractures were constant among age groups, with an even greater absolute risk reduction as age increases. This greater absolute risk reduction was explained by the age-related increase in fracture risk in the placebo group. Finally, also a very recent study showed that alendronate in patients older than 80 years who had a prior fracture was associated with a reduced hip fracture risk (HR 0.72; 95% CI 0.61–0.85) [92]. The reduction in hip fracture risk was maintained across all age quartiles and the absolute risk reduction at 5 years increased substantially by quartile of age. Moreover, adverse events were not more common in the higher age quartiles.

##### Risedronate

In 1999 and 2000, the VERT-trials demonstrated the efficacy of risedronate to prevent vertebral and non-vertebral fractures in postmenopausal women (mean age approximately 70 years) [103, 104]. In 2001, the effect on hip fracture risk was examined in the HIP-trial, of which one arm included postmenopausal women with a mean age of 74 years [93].

The other arm of the HIP-trial included 3886 women aged ≥80 years with a low femoral neck BMD or at least one non-skeletal risk factor for hip fracture (e.g., poor gait or a propensity to fall) [93]. After 3 years, no reduction in hip fracture risk was observed. Of note, the majority of the participants was selected based on non-skeletal risk factors [93]. A second analysis was a pooled analysis of the VERT-and HIP-trials in 1392 women with osteoporosis aged ≥80 years [94]. After 3 years, the risk of vertebral fractures was reduced by 44% (HR 0.56; 95% CI 0.39–0.81). The incidence of non-vertebral fractures was not significantly different.

The difference in benefit for vertebral versus non-vertebral fractures in the elderly might be explained by the fact that bisphosphonates impact on BMD, illustrated by the significant reduction of vertebral fractures. Bisphosphonates, however, do not impact on non-skeletal risk factors of fractures such as gait disturbances, impaired balance, and fall risk. These non-skeletal factors are of particular importance in the occurrence of non-vertebral fractures in the elderly, as they are more prone to falling. In contrast, vertebral fractures are often atraumatic, thus less influenced by non-skeletal risk factors. An additional explanation for this discrepancy between the older and younger population in preventing non-vertebral fractures, might be insufficient statistical power in the older age group.

##### Ibandronate

No data are available on the anti-fracture efficacy of ibandronate in the very elderly.

##### Zoledronic Acid

The HORIZON Pivotal Fracture Trial (HORIZON-PFT) showed that zoledronic acid is an effective therapy in postmenopausal women (mean age 73 years), with a reduction in the risk of vertebral, hip, and non-vertebral fractures [105]. In the HORIZON-Recurrent Fracture Trial (HORIZON-RFT), zoledronic acid reduced the risk of new vertebral and non-vertebral fractures in patients with a hip fracture (mean age 74.4 years) [106].

In 2010, a post hoc pooled analysis of both HORIZON trials that focused on women aged ≥ 75 years with osteoporosis was published (mean age 79.4 years) [95]. After 3 years, the incidence of vertebral and non-vertebral fracture was significantly lower in the treated group compared to the placebo group (HR 0.34; 95% CI 0.21–0.55 and HR 0.73; 95% CI 0.6–0.9, respectively). The benefit was comparable with the risk reduction in subjects <75 years in HORIZON-PFT and HORIZON-RFT. However, contrary to younger persons, the reduction in hip fracture risk did not meet statistical significance in patients ≥75 years. As mentioned above, possibly the sample size was not powered to detect hip fracture risk reduction in the older age group. Another explanation is the greater influence of non-skeletal risk factors for hip fractures with increasing age.

##### Denosumab

In women with a mean age of 72.3 years, denosumab has been established as a safe and effective therapy by the FREEDOM trial, with a significant risk reduction of vertebral, hip and non-vertebral fractures [107].

A post hoc analysis of FREEDOM was carried out in 2011 to evaluate the effect of denosumab in persons aged ≥75 years [96]. In this group (mean age 78.2 years), denosumab significantly reduced the risk of hip fractures by 62%, comparable with the risk reduction in the overall FREEDOM study population. Moreover, no significant difference in adverse events was observed in the older subgroup [96]. In 2012, another analysis of FREEDOM confirmed that denosumab reduces the risk of vertebral and non-vertebral fractures in subjects older than 75 years (RR 0.36; 95%CI 0.25–0.53 and RR 0.84; 95% CI 0.63–1.12, respectively) to the same extent as in younger subjects (RR 0.30; 95% CI 0.22–0.41 and RR 0.78; 95% CI 0.63–0.96) [97]. Moreover, treatment safety and efficacy of denosumab are not affected by renal function, so elderly with renal impairment will experience the same anti-fracture efficacy as patients with normal renal function [108]. However, since the use of denosumab is associated with a high rate of severe hypocalcemia in patients with advanced CKD, close monitoring and replacement of calcium and vitamin D is required to avoid the development of hypocalcemia in these patients.

Thus, denosumab is an effective therapy to prevent vertebral, non-vertebral, and hip fractures in the elderly, in contrast to bisphosphonates with no significant reduction in hip fracture risk by risedronate and zoledronic acid. As mentioned, this might be explained by lack of statistical power. However, it is tempting to speculate that this observation is due to the mechanism of denosumab, different from that of bisphosphonates, with distinct effects on cortical bone. Cortical porosity is indeed one of the main determinants of non-vertebral fracture risk, including hip fracture risk.

##### Strontium Ranelate

The anti-fracture efficacy of strontium ranelate was established by two trials, SOTI and TROPOS, in women with a mean age of 69.3 and 76.7 years, respectively [109, 110].

A preplanned pooled analysis of both trials was undertaken to confirm these results in patients older than 80 years [99]. After 5 years, the risk of vertebral and non-vertebral fractures was reduced by 31% (RR 0.69; 95%CI 0.52–0.92) and 27% (RR 0.73; 95%CI 0.57–0.95), respectively [99]. The numbers needed to treat were lower in women aged ≥ 80 years than in younger women. This is because a similar relative risk reduction in both age groups will avert more fractures in the older age group which has a higher baseline fracture risk [98]. Statistical significance for hip fracture risk reduction was not reached, but the analyses were not designed to quantify the reduction in hip fracture risk.

Thus, these analyses showed a significant reduction in the risk of vertebral and non-vertebral fractures in persons aged ≥ 80 years treated with strontium ranelate. However, the finding of an increased risk of cardiac events, including myocardial infarction, in addition to the already recognized risk of venous thromboembolism, has limited its use in clinical practice, although it remains a useful alternative in elderly with severe osteoporosis, unable to take other treatment and without cardiovascular contraindications [111].

##### Anabolic Therapy

Daily injections of teriparatide reduce the risk of vertebral and non-vertebral fractures, as shown in the Fracture Prevention Trial (FPT) in women (mean age 69.5 years) with a prior vertebral fracture [112].

A prespecified subgroup analysis of the FPT-study was undertaken in 2006 to investigate the effect of teriparatide in persons aged 75 years and older (mean age 78.3 years) [100]. In this subgroup, 5.2% in the teriparatide group and 15.1% in the placebo group had a new vertebral fracture after 19 months. Treatment-by-age interaction was not significant, indicating that the effect of teriparatide was not statistically different in younger and older patients. Also for non-vertebral fractures, the treatment-by-age interaction was not significant. There was no significant difference in the safety profile between younger and older participants. So, age does not affect the safety and efficacy of teriparatide in preventing fractures. The major disadvantage, however, is the daily subcutaneous administration which may be a burden for elderly.

Also for abaloparatide, a novel 34-amino acid PTH-related peptide (PTHrP) which significantly reduced the risk of new vertebral and non-vertebral fractures in the Abaloparatide Comparator Trial in Vertebral Endpoints (ACTIVE), there was a consistent fracture risk reduction across different age groups (<65 vs. 65 to <75 vs. ≥75 years) [113].

## Conclusion

In old age, osteoporosis and osteoporotic fractures tend to occur in a particularly frail subset of the population. Treatment of osteoporosis is of particular concern in the elderly because of the substantial burden of osteoporotic fractures in terms of morbidity, mortality, and economic cost. Calcium and vitamin D is an essential component in the management of osteoporosis in old age. Adding osteoporosis treatment reduces the risk of fractures even more, at least in older individuals with documented osteoporosis and at least for vertebral fractures and maybe also for hip fractures. In frail elderly with documented osteoporosis, treatment may even be more effective than in younger patients.

## General Conclusions

In this consensus paper, we reviewed the evidence about the evaluation and management of osteoporosis in frail persons. This is of particular concern because of the major burden of osteoporotic fractures in terms of morbidity and mortality in frail individuals such as patients with AN, dialysis and cancer patients, persons with sarcopenia, and the oldest old.

AIs, ADT, and HSCT in cancer patients are associated with increased bone loss and fracture risk, as are dialysis status, old age, and AN. Sarcopenia is also associated with low BMD. Low muscle and bone mass results in a decrease in physical activity, ultimately leading to osteosarcopenic obesity.

Therefore, individuals treated with AIs, ADT, and HSCT, as well as elderly and/or sarcopenic individuals and patients with AN should have their bone health monitored for fracture risk with DXA. Until recently, DXA was not recommended in dialysis patients, but this may change in future as recent data suggest that DXA may also predict fractures and maybe even mortality in dialysis patients. In the FRAX algorithm, age and BMI, but not anticancer treatments, dialysis status, and sarcopenia are considered as specific risk factors. Therefore, the effect of these later conditions on fracture risk may be underestimated by this algorithm.

In all these frail individuals, a healthy lifestyle should be recommended, consisting of physical exercise (except in AN), adequate calcium and vitamin D intake, and certainly in persons with AN and osteosarcopenic obesity, recovering a normal BMI. Furthermore, in patients on AI and ADT, bisphosphonates and denosumab administered at doses similar to the treatment of postmenopausal osteoporosis are recommended to prevent bone loss and reduce fracture risk. After HSCT, also testosterone may be considered in hypogonadal men and MHT in menopausal women. AN adolescents may also be treated with MHT, while antiresorptives and/or anabolics are only indicated in adults, not adolescents, with AN. Similarly, in elderly, antiresorptive and/or anabolic treatment is indicated to prevent osteoporotic fractures. As compared to younger individuals, elderly have a higher absolute risk reduction because of their higher baseline fracture risk. In dialysis patients, there are few studies, although some data suggest a positive effect of bisphosphonates, in lower dosages than used in the general population, and of denosumab which is, however, associated with a higher risk of severe hypocalcemia. Antiresorptives should be avoided in dialysis patients with low bone turnover, in whom teriparatide may be of theoretical interest. Finally, more research is needed to develop pharmacological therapies that have a concomitant effect on muscle and bone.

To conclude, frail individuals are at risk of bone loss and fractures. Therefore, in these patients, it is advised to regularly monitor bone health by DXA or, if available, other techniques such as QCT. FRAX may not always adequately predict fracture risk. Strategies to prevent bone loss and fractures include advising a healthy lifestyle as well as initiating specific osteoporosis therapy. Further research is needed to determine the role of new osteoporosis medication in frail individuals as well to develop medication that target not only bone, but also concomitant affected systems such as muscles and the cardiovascular system.
